# The Need for a Prevention Program for Child Sexual Abuse in Brazil: A Report of Shortfall Care to Pedophilia and its Critical Consequences

**DOI:** 10.1007/s40653-023-00586-2

**Published:** 2023-12-02

**Authors:** Gabriel Furian, Klaus Michael Beier, Karine Schwarz, Dhiordan Cardoso da Silva, Dorit Grundmann, Maria Inês Rodrigues Lobato

**Affiliations:** 1 Graduate Program in Medical Sciences: Psychiatry, Universidade Federal do Rio Grande do Sul, Porto Alegre, RS Brazil; 2 Institute of Sexology and Sexual Medicine, Charité – Universitätsmedizin Berlin, Berlin, Germany; 3 Gender Identity Program at Hospital de Clínicas de Porto Alegre, Porto Alegre, Rio Grande do Sul Brazil; 4 Hospital de Clínicas de Porto Alegre, Rua Ramiro Barcelos, Porto Alegre, 2350, Rio Grande do Sul 90035-903 Brazil; 5 Postgraduate Program in Medical Sciences: Endocrinology, Faculty of Medicine, UFRGS, porto alegre, Brazil

**Keywords:** Pedophilia, Child sexual abuse, Patricide, Case report, Prevention and treatment strategies in Brazil

## Abstract

From a case report of a person with pedophilic disorder, this paper focuses on the issue of pedophilia, child sexual abuse, and the need for specific prevention and treatment strategies in Brazil. It seems inevitable to increase awareness for this topic within the mental health care system to protect children and reduce the risk for sexual offense in individuals at-risk. This is a case report of an individual, known by medical-psychiatric and forensic facilities for a past history of patricide, who revealed his pedophilic fantasies and behavior belatedly. To assess a pedophilic disorder and screen for other paraphilic contents, a screening questionnaire and clinical interview were used during the patient hospitalization in 2020 for a proper evaluation of sexual history and past offending behaviors. A review of the literature on pedophilia prevention programs was also carried out. WW is a middle-aged man admitted to a psychiatric unit for a severe episode of major depressive disorder and at risk of suicide. During recovery, he reported pedophilic fantasies and behaviors in his life. Sexual fantasies involving children and actual sexual offenses have remained unknown to mental health professionals and unreported to legal authorities. WW’s case alarmingly emphasizes the need for the training of health care professionals and for preventive strategies in Brazil for those who are at risk of engaging in offending sexual behaviors in a combined and intensive effort to protect children from sexual offense.

## Introduction

Pedophilia is recognized by the World Health Organization (WHO) as a medical condition, described as a paraphilia [“The term paraphilia denotes any intense and persistent sexual interest other than sexual interest in genital stimulation or preparatory fondling with phenotypically normal, physically mature, consenting human partners” (American Psychiatric Association APA, 2013)]. Pedophilia is defined as a sexual preference/interest for the body forms of children. Sexual preference generally manifests during adolescence and remains relatively stable thereafter. However, some medical measures such as Cognitive Behavior Therapy, associated or not with hormone therapy, have been applied in some cases with some positive results for control of sexual impulses (Tenbergen et al., 2015).

CSA is the criminal act of sexually offending children. Non-pedophilic offenders who use children as a surrogate for an adult partner, do not necessarily have a sexual preference for children and consequently do not suffer in this regard (Beier, 2019, 2021). In other words, not all CSA offenders have a pedophilic disorder. Note: not all persons with pedophilia act out on their urges (Knack et al., 2019).

Unfortunately, we are currently unable to identify potential offenders who have pedophilic disorder, prior to the occurrence of sexual abusive behavior. Interestingly, empirical data suggests that pedophiles have higher levels of co-morbidity and distress due to problems associated with their sexual preference, and they are more likely to seek treatment than other sexual offenders. One avenue to identify a potential CSA person is by organizing medical services specialized in giving them opportunities for treatment. However, little has been done to offer such services, though with some exceptions in Europe and North America (Beier et al., 2009, 2015; Konrad et al., 2017; Knack et al., 2019; Beier, 2019; Fedoroff, 2020).

It is well known that most children who have suffered CSA, will develop many psychiatric symptoms such as anxiety, depression, or unstable personality traces, causing irreparable damage for present and future individual psychosocial performance, rendering it essential to provide programmes for the prevention of CSA (Hem et al., 2013; Hailes et al., 2019).

For the last 20 years in Brazil, many child protection measures have been implemented, such as the training of mental health and school teams, to identify any signs of children in psychological distress who may have been victims of abuse. Also, since 2009, the Brazilian criminal legislation for CSA has become very rigorous, resulting in many more penitentiary sentences. Unfortunately, this has not resulted in the reduction of victims of sexual abuse. It is our concern in Brazil that we need to do more, since we do not have any specialized treatment services. We plan to execute this soon!

The aim of the following case report is to call attention to this issue in our country. A man served a 15-year sentence for parricide without having been identified with pedophilic disorder, either by the public judiciary or the mental health system. We hope this particular case will stimulate different professionals to organize medical services that can prevent new CSAs.

## Methods and Ethical Considerations

The screening questions for paraphilic interests were based on the “Troubled Desire” project, a web-based program that offers anonymous and confidential self-assessment and self-management training modules for individuals with sexual interest in children (Schuler et al., 2021). The English version of this questionnaire was provided from personal correspondence with the project coordinator of the German “Troubled Desire” program. The questionnaire was translated and adapted from the English version to Brazilian Portuguese by two bilingual psychiatrists, and later reviewed by a native English speaker. There were many steps before reaching the final Brazilian Portuguese version which is still in the validation process. However, for the purpose of this case study, the in-process version was considered sufficient for clinical use and evaluation of the respective information on the patient’s sexual experiences and behaviors.

The information given in this article has been chosen to preserve the patient’s identity and confidentiality. The patient signed the informed consent form, allowing the use of his data for this case report.

### Case Report

WW, a middle-aged Brazilian male with a past psychiatric history of depression and suicidal thoughts was interviewed in 2020, during his stay in a psychiatric inpatient due to his risk of suicide. Interview and evaluations were carried out by a team of researchers with experience in the subject, without prior judgments. These conditions probably favored better patient care and comfort in answering delicate questions related to their sexuality.

### History of Present Illness

During the last six months before his admission to the hospital, WW reported gradually feeling sad and experiencing a lack of motivation to keep his home activities alongside a lack of appetite and hypersomnia. He reported command auditory hallucination telling him to commit suicide. WW has been followed-up by a mental health team since 2018 and has been taking six ampoules of haloperidol decanoate per month – actual intake of the medication was not monitored.

He did not finish elementary school and over the years had several jobs in southern Brazil. He has been retired for over a decade due to his mental health condition. He lived alone in a small house for several years and was expelled by the community due to his suspicious CSA history. Thus, he was homeless in the days prior to his admission to the psychiatric inpatient unit.

WW had a history of alcohol use disorder (at least one bottle of wine per day) and nicotine misuse (two packs of cigarettes per day). He started consumption of both in his twenties. He had emphysema and diabetes mellitus type 2, but he does not adhere to treatment.

### Previous History of Mental Illness

WW has had other mood episodes in the past. During one of these episodes, he had planned suicide by setting his house on fire with cooking gas. He reported having been hearing multiple auditory hallucinations since his adolescence, but only started seeing a psychiatrist in his twenties, after murdering his father with a gun. He claimed to be hearing command auditory hallucination that guided him to commit patricide. Due to this crime and his mental condition, WW spent more than a decade at a forensic unit (FU).

Nevertheless, his pedophilic behavior became known only during the current admission to the hospital. WW said he was sexually attracted to his father’s stepdaughter and maybe “he fell in love with her,” and he may not have tolerated the way his father treated the child (“my father put the little girl on his lap… he ran his hand over her several times”). This “little girl” WW mentioned was about 11 years old, in the very beginning of her puberty.

### Psychosocial Profile

WW was raised by his biological parents. He reported having a good relationship with his family (mother, father, and siblings) until the age of ten, when he found out that his father was cheating on his mother. She died before his twenties and soon after, his father married another woman, who already had a daughter. He is single and has never had a relationship, nor children. WW considers himself a “straight” man (i.e., sexually oriented towards women), but admitted having difficulties to form relationships and have sexual contact with women he was interested in. He perceives himself as “ugly, shy, and not interesting.”

### Hospitalization

His mood and psychotic symptoms improved by taking fluoxetine 40 mg and haloperidol 10 mg and he no longer mentioned having suicidal thoughts. He was collaborative in the interviews. When asked about his sexual fantasies and history of offenses, he was embarrassed, but we assured him medical confidentiality and he answered sincerely. WW tended to avoid discussing these topics since he had many troubles and difficulties during his life. He considers that these pedophilic behaviors have worsened his capability of establishing relationships.

Due to his history of alcohol use disorder and an impression of some cognitive impairment by the medical team, an MRI of the skull/brain was performed, and his IQ was assessed by a psychologist performing the Wechsler Adult Intelligence Scale (WAIS-IV). The MRI showed diffuse reduction of the encephalic volume, more evident in the supratentorial compartment, and an increase in the ventricular cavities size, not proportional to the encephalic volume dimension. There was some supratentorial microangiopathy and caudal insinuation of the right cerebellar tonsil. Contrasting with the MRI, the evaluation of his intelligence showed a global IQ score of 93 (percentile 32), with an execution IQ of 79 (percentile 8) and a verbal IQ of 110 (percentile 75).

He was also evaluated by a forensic psychiatrist who issued the clinical recommendation that the patient requires a structured environment to organize himself and to accomplish his psychiatric treatment, for example, in a therapeutic residential setting. Considering his psychiatric history, ongoing support and attention of a mental health team may reduce the risk of worsening symptoms and unsafe behavior and ensure psychopharmacological medication compliance. This forensic assessment has no mention of antisocial traits.

He showed a critical clinical situation (severe emphysema, diabetes, hypertension), history of poor adherence to clinical and psychiatric treatment, social vulnerability, and lack of family support. After 35 days of psychiatric hospitalization, WW presented a more stable mental state and was transferred to a Public Therapeutic Residential Complex for constant monitoring by the health care team.

### Sexual History

During the clinical interview, WW reported to be sexually aroused by either prepubescent or early pubescent girls, with a dominant attraction to girls at Tanner stage II. Besides the pedophilic/hebephilic sexual interest, he is also attracted to adult women, but to a lesser extent. Sexual arousal towards boys or adult men was not reported, but later in the interview, WW described sexual encounters with an adult male when he was first admitted to a psychiatric unit. WW reported to be aware of his sexual interest in younger girls and adult women since he was 15 years old.

During masturbation, he fantasizes about children doing regular activities such as playing, but he also fantasizes about girls in provocative poses or even about girls having sexual intercourse with him. He reported to be sexually aroused by the idea of showing his penis to girls. He denied ever watching pornography and has never had access to the internet. In the past, he made use of pornographic magazines (like *playboy*) and sometimes visited “porn cinemas.”

Directly asked about past problematic sexual behaviors involving female minors, WW denied any sexual approach and claimed that he only liked to watch children play. However, during the interview, WW reported more than 50 cases of child sexual abuse against girls between the ages of three and ten, when he was between the ages of 15 and 33. The last time was during a home visit while he was housed at the FU.

Sexual interactions with girls involved exhibitionistic acts of presenting his genitals, touching a child’s buttocks in playful situations, rubbing his penis on a child’s buttock or vulva and oral contact with the vulva, without vaginal or anal penetration. The children he sexually abused were mostly girls from his neighborhood and he convinced them to come to his home by offering candy or money. He also reported to be sexually aroused by the fantasy of rubbing his genitalia on women’s buttocks when he is in public transportation.

## Discussion

WW has clearly shown a sexual preference towards prepubescent and early pubescent girls since he was around 15 years old. There is a clear sexual interest towards the prepubescent female body which motivated him to seek children (followed by the first offenses unreported to legal authorities), regardless of any other reason such as alcohol misuse or the presence of any psychotic symptoms. He last committed the offense during a home visit when he was living at the FU, while supposedly adhering to the treatment – revealing the diminished inhibitory control for his sexual urges.

This case draws our attention to the fact that, despite the long-term contact with the public health care system, WW’s intensive CSA behavior went almost unnoticed throughout the period he was at the FU. He only disclosed this troubled sexual behavior during his recent psychiatric hospitalization, where he had been admitted due to depression after he was expelled from his neighborhood because of his sexual offenses against children.

The pedophilic disorder influenced his patricide history due to perception that his father was sexually inappropriate with the stepdaughter/patient’s stepsister. Considering the follow-up of this case, WW did not meet the diagnostic criteria for schizophrenia, schizoaffective disorder, or bipolar disorder, thus ruling out that the described hallucinatory phenomenon would allow for a milder sentence in the Brazilian judicial system.

Furthermore, this case report aims to alert the Mental Health Care System to CSA and the use of CSAI associated with pedophilia and to the importance of discussing and bringing to light this very controversial topic. The CSA might interfere with children’s growth and development (Foster & Carson, 2013; Goodman et al., 2010). It has also been linked to several maladaptive health behaviors, and poor social, mental, and physical health outcomes (Putnam, 2003; Irish et al., 2010). The scientific research focuses mainly on the psychiatric and psychotherapeutic approach concerning victims of CSA whereas the offender’s treatment is mostly considered an academic shortfall (Tenbergen et al., 2015). Therefore, primary and secondary prevention strategies against minor sexual abuse are definitively insufficiently considered. Identifying high-risk individuals who may have a pedophilic/hebephilic disorder and offering them therapeutic strategies and support to control this kind of behavior, should be just as important as treating its critical consequences.

Sexual violence against children and adolescents has been given political importance in Brazil after the child and adolescent statute was institutionalized in 1990 (Estatuto da Criança e do Adolescente e outras providências, 1990). Based on this movement, several spheres of Brazilian society have engaged in the process of defending rights and developing public policies for this purpose (Paixão & Deslandes, 2010). In 2009, with law no. 12,015, the Brazilian Penal Code standardized a strict punishment for sexual abusers considering that any abuse would culminate in a minimum of eight years in a closed regime (Crimes contra a dignidade Sexual e crimes contra a liberdade sexual, 2009). These social, medical, and legal upgrades were associated with increase in the notification of cases of sexual violence (Secretaria de Vigilância em Saúde, 2018) and in the incarceration of sexual abusers, but unfortunately no evidence shows a reduction in numbers of assaults.

These political changes seek to ensure children’s protection, offering training to mental health assistance teams to identify indicators of traumatization from sexual abuse in children. However, nothing has been done to identify early high-risk individuals who may commit CSA offenses and consume CSAI. For this, we must focus on supporting research on sexual offenders, assessing the pathophysiology—as well as pharmacological and psychotherapeutic treatment approaches in the context of pedophilic sexual preference and behaviors—and finding strategies for preventing CSA and the use of CSAI in Brazil.

In conclusion, primary prevention against CSA, which includes the identification, diagnosis, and treatment of individuals with a pedophilic sexual interest, is considered the most effective approach.

## Conclusion

Given the positive results of using the screening questionnaire based on the “Troubled Desire” project and the patient’s disclosures, the authors recommend the tool especially for assessing paraphilic sexual interests and behaviors, with a focus on pedophilia. Mental health professionals should take the opportunity of using the questionnaire for their clinical evaluations.

WW is a typical example of those patients who did not have the chance to effectively communicate pedophilic behaviors despite having had contact with many mental health professionals over the years.

Finally, this case report of WW highlights the medical approach that has been previously inefficient in terms of early (or even late) identification of pedophile individuals and its serious negative impact on preventing CSA. The current medical approaches were not able to develop research on appropriate treatments for potential offenders who may eventually perpetrate the abuse.
